# Neonates in the Intensive Care Unit: Maternal Health-Related Quality of Life and Depression After Term and Preterm Births

**DOI:** 10.3389/fped.2021.684576

**Published:** 2022-01-06

**Authors:** Eva Mautner, Christina Stern, Alexander Avian, Maria Deutsch, Wolfgang Schöll, Elfriede Greimel

**Affiliations:** ^1^Department of Obstetrics and Gynecology, Medical University of Graz, Graz, Austria; ^2^Institute for Medical Informatics, Statistics and Documentation, Medical University of Graz, Graz, Austria

**Keywords:** health-related quality of life, depression, preterm birth, neonates, intensive care unit

## Abstract

**Background/Objective:** To examine maternal physical and mental health-related quality of life (HRQoL) and depression after early and late preterm and term births in the early postpartum period.

**Method:** In a prospective pilot study, three groups of women whose newborns had to be treated in the neonatal ward during the immediate postpartum period were established and compared with each other: 20 women with extremely to very preterm birth, 20 with moderate to late preterm birth and 20 women with term birth. All participants completed the Short Form-12 Health Survey (SF-12) to measure HRQoL, and the Edinburgh Postnatal Depression Scale (EPDS) to detect depressive symptoms combined with independently developed questions to evaluate anxiety and psychological distress.

**Results:** Maternal psychological HRQoL was significantly worse in the very preterm birth group compared to moderate to late preterm birth (*p* < 0.001) and full-term birth groups (*p* = 0.004). There were no differences between the birth groups in depressive symptoms (*p* = 0.083), anxiety (*p* = 0.238), perceived stress (*p* = 0.340) and the general psychological distress values (*p* = 0.755). In the EPDS, the depression screening instrument 30 to 65% were beyond the cut-off-value to detect major depression.

**Conclusions:** During the early postpartum period, an extensive medical care focussing on acute stress, HRQoL parameters and depression may be a good step to improving maternal well-being.

## Introduction

Worldwide preterm births and the treatment of neonates in the neonatal ward have an impact on the emotional well-being of women. Preterm births are defined as occurring before 37 weeks of gestation. Under-five-year-olds are at the forefront of child mortality worldwide and are associated with serious morbidities and long hospital admissions (1, 2). There is a differentiation between early gestational age infants, in which the risks of mortality and morbidity are much higher than in late preterm infants and infants born at term (3, 4). Several psychological reactions to preterm deliveries include parental acute stress, anxiety, depression, along with poorer family functioning (5–13). Postpartum depression (PPD) is a disorder which frequently affects women in the postnatal period and has consequences for the infant and the whole family. Since there are several treatment options for PPD (14), the recommendation for obstetric care providers is to accomplish an assessment of mood and emotional well-being during the postpartum period for each patient (15, 16). Another option to evaluate mood or well-being besides depression is HRQoL, which includes various dimensions of health and functioning. According to the World Health Organization (WHO) HRQoL represents “an individual's perception of their position in life in the context of the culture and value systems in which they live and in relation to their goals, expectations, standards and concerns (17).” HRQoL scores are implemented in several settings, such as conducting research, monitoring, and reporting or establishing quality improvement strategies. HRQoL is inspired by personal and environmental factors and their interactions, and can be enhanced through individualized support and personal growth opportunities (18–20).

The purpose of this study was to explore differences in maternal HRQoL and depression of mothers after preterm birth whose newborns had to be treated at the NICU, distinguishing between (1) very preterm and (2) moderate to late preterm, compared to (3) term births. We hypothesized that early preterm birth is associated with more depressive symptoms and decreased HRQoL compared to moderate to late preterm and term birth during the early postpartum period.

## Materials and Methods

This prospective questionnaire pilot survey was conducted in the Obstetrics Department of a major teaching hospital in Austria, which serves as a tertiary referral center. The study was carried out in agreement with the guidelines recommended by the World Medical Association of Helsinki and was endorsed by the local Ethical Committee (Nr.: 28-269 ex 15/16). There were no monetary or other incentives for women's contribution. Recorded informed consent was obtained from all the women.

Inclusion criteria were German-speaking women who had undergone preterm or term birth according to the intended groups (extremely to very preterm 23+0 until 31+6, moderate to late preterm 32 + 0 to 36 + 6 gestational week) or women who had full-term births (>37 weeks of gestation) and were separated from their neonates because of the newborns' need for intensive care at the neonatal ward (NICU). Exclusion criteria were the inability to speak or understand the local language. Inpatient women were contacted during their postnatal stay in the hospital from December 2016 to December 2018. After agreeing to participate, women given the informed consent form and the questionnaire set. Minimum to maximum intended time interval from birth to study inclusion was three to 10 days postpartum.

Ten persons (out of 70) refused to take part in the study: one woman of the very preterm birth group, three of the moderate to late preterm group and six of the term birth group. We compared accessible characteristics between participants (*n* = 60) and non-participants (*n* = 10) and found no statistically significant differences on age (*p* = 0.932) and study groups (*p* = 0.199).

### Instruments (Questionnaires)

Women's HRQoL in the past month was evaluated by the German edition of the Short-Form-12 Health Survey (SF-12). It is a worldwide standardized instrument to measure self-assessed HRQoL (21–23). Twelve questions measure the general health perception and general mental condition, physical and social functioning, role limits due to physical health problems or emotional problems and queries on vitality and bodily pain. Scoring guidelines are available to create standard values with a mean of 50 and a standard deviation of 10 (22, 23). The standardization enables cross-cultural comparison (22, 24). The reliability of all SF-12 scales was 0.88(22).

The Edinburgh Postnatal Depression Scale (EPDS) was applied to determine postnatal depression (25). It is an internationally established 10-item questionnaire. Women rate how they have felt during the past seven days. Each item is scored from 0–3 (resulting range 0–30). In many countries the EPDS is routinely administered to detect postnatal depression. For Austrian perinatal and postnatal women, a cut-off value of 10/11 is suggested with a sensitivity of 0.87 and specificity of 0.87 to detect all cases of major depression (26). We chose this depression screening instrument because it is widely endorsed and gives a clear overview.

To minimize the completion time for the participating women, postnatal stress and anxiety was measured with short independently developed single Likert scales. Women were asked how much stress and anxiety they have perceived during the previous 3 days (from 0%, which is not at all, to 100%, which is very much). General psychological distress was assessed with a single Likert scale from zero, which is not at all, to five, which is the most distress experienced.

On a separate sheet we asked about demographic and medical data including questions about number of children, maternal age, gestational week at birth, maternal hospitalization before birth, weight of the newborn, hours of sleep during the last day, perceived participation and control, education level, living situation, employment status and financial situation.

### Statistical Analysis

Continuous data are shown as median and interquartile range (IQR) or mean and standard deviation. Categorical data are presented in absolute and relative frequencies. Differences among the three preterm groups were computed with Kruskal-Wallis test or ANOVA for continuous data and Fischer's exact test or χ^2^—Test for categorical data. Since we performed a pilot study *p*-values of *post hoc* analyses were not corrected for multiple comparisons. A *p*-value < 0.05 was considered statistically significant. Statistical evaluations were implemented using IBM SPSS 24.0 (IBM Corp., Armonik, NY, USA).

## Results

### Sample Characteristics

The study group consisted of 60 women, who gave birth preterm or at term within the last 9 days. The medical reasons for extremely to very preterm births were progressive preterm labor (i.e., preterm contractions, preterm rupture of membranes, prolapsing amniotic membranes, (*n* = 9), pre-eclampsia (*n* = 6), and complicated twin pregnancies (*n* = 5). The medical reasons for moderate to late preterm birth were progressive preterm labor (i.e., preterm rupture of membranes, preterm contractions, *n* = 8), complicated twin pregnancies (*n* = 7), pre-eclampsia or HELLP-syndrome (*n* = 2), fetal hydronephroses (*n* = 1), placenta praevia (*n* = 1) and fetal bradycardia (*n* = 1). The term infants were treated in the neonatal ward because of perinatal infection (*n* = 9), infant respiratory distress syndrome (*n* = 5), fetal hydronephroses (*n* = 1), fetal anal atresia (*n* = 1), intrapartum fetal bradycardias (*n* = 1), cleft lip and palate (*n* = 1), small-for-dateness (*n* = 1) and congenital ichthyosis (*n* = 1).

As shown in Table 1, median maternal age of the whole sample (*N* = 60) was 32.0 years. The median birth weight of the infants was 1,953 grams (range from 550 to 3,985 grams), and the median week of gestation at birth was 34 weeks (range from 25 to 42 week of gestation). The intended time interval between birth and day of the survey was day three to 10 postpartum. On average, women responded on the fifth day (range from three to nine). All maternal information and characteristics are presented in Table 1.

**Table 1:** Demographic and medical characteristics of the study population (*N* = 60).

**Maternal characteristics**	**Mean (SD)**	**Range**
Maternal age	31.45 (5.2)	17–43
Number of children	1.4 (0.7)	1–4
Gestational week at birth	34.2 (5.1)	25–42
Maternal hospitalization before birth	12.6 (15.2)	0–54
Weight of the infants	2,076.4 (1,024.5)	550–3,985
Hours of sleep during a day	5.86 (1.39)	3–10
Perceived participation and control^*^	43.15 (29.96)	0–100
	Numbers	Percentages
Maternal highest educational level		
Primary school	3	5
Secondary school	17	28.3
Higher education, not university	19	31.7
Higher education, university	21	35
Living situation		
With children	2	3.3
With partner and children	34	56.7
With partner, children and grandparents	7	11.7
With partner	17	28.3
Employment status		
Not employed	7	11.7
Full-time employed	36	60
Part-time employed	17	28.3
Financial situation		
Very satisfying	12	20
Satisfying	44	73.3
Less satisfying	4	6.7

*SD, standard deviation*.

* *Scale from 0 to 100*.

Demographic data did not differ between study groups, including age, number of children, mode of delivery, hours of sleep during the last day, perceived participation and control, maternal education level, living situation, employment status and the financial situation (see Table 2). However, regarding the time of hospitalization before birth the groups varied significantly (*p* = 0.015). Women with moderate to late preterm births spent significantly more days (median: 16 days, IQR: 6-32) in the hospital before birth compared to mothers of the very preterm (4 days, IQR: 1-19; *p* = 0.025) and mothers of the term group (median: 2, IQR: 1-7; *p* = 0.009).

**Table 2:** Differences between the three birth groups in psychological outcomes and maternal characteristics.

**Psychological outcomes**	**Early preterm <32 weeks**	**Moderate to late preterm** **32–37 weeks o**	**Term birth >37 weeks**	
	**Mean ± SD** **Median (IQR)**	**Mean ± SD** **Median (IQR)**	**Mean ± SD** **Median (IQR)**	***p***
SF-12 Physical component summary (PCS)	41.62 ± 7.23 ^A^	35.71 ± 7.43 ^B^	37.02 ± 6.90	0.046^*^
SF-12 Mental component summary (MCS)	36.82 ± 6.32 ^A.^	46.58 ± 7.20^.B^	43.31 ± 6.20^.B^	<0.001^**^
EPDS Depression	11 ± 6.	12 ± 6.	8 ± 6	0.083
Perceived stress during the last three days	70 (50–85)	55 (35–75)	70 (70–80)	0.340
Perceived anxiety during the last 3 days	80 (50–90)	55 (35–80)	80 (60–80)^A.B^	0.238
General psychological distress	3 (2–4)	3 (2–4)	3 (3–4)	0.755
Maternal characteristics				
Maternal age (years, range)	30.55 (21–38)	31.8 (24–43)	32 (17–42)	0.642
Number of children (median IQR)	1 (1–2)	1 (1–2)	1 (1–2)	0.666
Hospitalization before birth (Median IQR days)	4 (1–19)	16 (6–32)^***^	2 (1–7)^***^	0.015
Weight of the infants (Median ± SD)	1,006 ± 342^***^	1,972 ± 294^***^	3,313 ± 464^***^	<001
Mode of birth				0.081
Spontaneous vaginal	5	4	9	
Planned cesarean	7	13	7	
Emergency cesarean	8	2	4	
Hours of sleep during a day (Median IQR)	6 (5.5–6)	6(5–7)	5 (4–6)	0.288
Perceived participation and control (Median IQR)	40(10–60)	50(20–70)	40(20–70)	0.441
Maternal educational				0.568
Primary school	2	1	0	
Secondary school	8	5	4	
Higher education, not university	5	6	8	
Higher education, university	5	8	8	
Living situation				0.629
With children	1	1	0	
With partner and children	10	10	14	
With partner, children and grandparents	2	4	1	
With partner	7	5	5	
Employment status				0.528
Not employed	4	1	2	
Full-time employed	10	12	14	
Part-time employed	6	7	4	
Financial situation				0.589
Very satisfying	5	3	4	
Satisfying	13	17	14	
Less satisfying	2	0	2	

* *Statistically significant group differences at the 0.05 level between A and B*.

** *Statistically significant group differences at the 0.01 level between A and B*.

*** *Significant difference between the groups*.

### Main Results

Comparisons of maternal mental HRQoL between the three birth groups (very preterm to term) demonstrated statistically significant differences during the early postpartum period (presented in Table 2). Women from the very preterm group showed significantly decreased mental HRQoL (36.8 ± 6.3) compared to those in the moderate to late preterm group (46.6 ± 7.2; p < 0.001) and term group (43.3 ± 6.2; *p* = 0.004). Correspondingly, comparisons of physical HRQoL between the three subgroups presented also significant differences (*p* = 0.046). *Post hoc* analyses showed that women in the moderate to late preterm group (35.7 ± 7.4) had significantly lower physical HRQoL compared to those of the very preterm group (41.6 ± 7.2; *p* = 0.020).

Regarding depressive symptoms we found no significant differences among the three birth groups (*p* = 0.083). However, 45 % of the women in the very preterm group presented an at-risk EPDS score above the cut-of-value of 10 (11, ±6), 65% in the moderate to late preterm birth group (12, ±6) and 30% of women giving full-term birth (8, ±6).

Women showed no differences in anxiety level during the last 3 days (*p* = 0.238). Additionally, women did not differ regarding acute stress (*p* = 0.340) and the general psychological distress values (*p* = 0.755).

## Discussion

### Key Results

The goal of this questionnaire survey was to investigate HRQoL and depression in women after being separated from their newborns immediately after birth in two preterm birth groups compared to mothers of hospitalized full-term neonates. As hypothesized, the study results show that maternal psychological HRQoL was significantly worse in the early gestational birth group compared to moderate to late preterm birth and full-term birth group. Remarkably women with moderate to late preterm births showed significantly the worst physical HRQoL compared to others with very preterm births and term births. The whole study group showed high at-risk EPDS scores. Unexpectedly, women in the moderate to late preterm group showed the highest percentage of depressive symptoms with 65%. All women experienced similar high anxiety, acute stress, and general psychological distress values.

### Interpretation

Early prematurity has, as expected, a serious effect on women's mental HRQoL in the early postpartum period. Parents, especially mothers, are usually not prepared for a preterm birth as it often occurs suddenly, and women describe this event as a stressful and sometimes even traumatic experience. The arrival of an unexpected early premature infant, as described earlier, leads to maternal feelings of helplessness and fears that she will not be able to care and protect her newborn (27). Furthermore, women are confronted with very long hospital stays of their infants at the NICU, sometimes lasting months, and this is accompanied by alterations in normal life (work, family life, financial situation). During the first days women receive information from different sources about their newborns' health and perspectives, for example about risks of neurodevelopmental or general developmental delays, motor, visual, and hearing problems or learning difficulties (1, 2, 12). Consequently having a very preterm infant and apprehensions about short-term and long-term problems increases maternal stress and anxiety as found before (12, 27) but also alters mental HRQoL.

According to maternal physical HRQoL, our study specifies that moderate to late prematurity was accompanied with most impaired physical HRQoL. We speculate that the prenatal hospitalization of women of the moderate to late preterm group has an apparent influence on physical mobility. Women of this group were hospitalized longer prenatally than the women in the other groups to prevent early prematurity (on average 16 days). Most of them were confronted during pregnancy with the diagnosis of a severe pregnancy complication, such as hypertensive disorder, complicated twin pregnancies, intrauterine growth restriction, preterm premature ruptured membranes or cervical insufficiency. Receiving the diagnosis of a high-risk pregnancy combined with the longer inpatient treatment seem to be serious stressors for women and caused the lowest women's physical HRQoL and more depressive symptoms. However, the prevention of early prematurity was possible in this group, since all of them delivered between the 32nd and 37th week of gestation. Therefore, it appears reasonable that mental HRQoL was better in these women compared to women after extremely to very preterm births.

Maternal at-risk depression rates were high in our whole study population. It ranged from 45% in the early preterm group, 65% in the moderate to late preterm group and 30% in the term birth group. Correspondingly, other studies found that mothers of infants born prematurely have high PPD rates (28–40%), particularly in the early postpartum period in the NICU (7, 13). Roomruangwong et al. (13) found that a history of depression in first week postpartum was associated with depression 4–6 weeks postpartum among mothers of infants at the NICU. Therefore it is important how women feel immediately after preterm and term births. Vigod et al. (7) describes in their systematic review that “sustained depression was associated with earlier gestational age, lower birth weight, ongoing infant illness/disability, and perceived lack of social support.” In general, postpartum depression (PPD) is the most frequent complication in all childrearing women. The general expected prevalence of PPD ranges according to Steward and Vigod (14) from 6.5 to 12.9 percent.

There are several studies which give inconsistent findings about how the mother-infant relationship develops following a preterm birth, concluding that the mother-preterm infant relationship is complex (27). Recent promotions of family-centered neonatal care seems to be promising. Both parents are inspired to be present and have the opportunity to be involved in the care of their newborns (27–29). However first it seems to be important to reestablish the maternal emotional well-being. Brecht et al. (5) published a review about the effectiveness of therapeutic behavioral interventions for parents of premature babies. The authors found a trend “toward early, brief interventions that are theoretically based, specifically target parent trauma, and utilize cognitive behavioral techniques.” It might be a good approach to provide maternal well-being and HRQoL immediately after preterm births, or after the diagnosis of severe pregnancy complications earlier during pregnancy. Women need to understand their first stress reactions after severe pregnancy complications and preterm births. Early brief intervention can lead to more competence, reduce depressive symptoms, and improve HRQoL. Women could be better prepared for the adventure of having a preterm or a term baby at the NICU. They need to understand their reactions to the NICU environment and their infant's situation to develop coping strategies and take an active role in care. Brief interventions will improve self-efficacy, will reduce depressive symptoms and improve maternal physical and mental HRQoL.

### Strengths and Limitations

The strength of the survey is the insight it gives into maternal postpartum HRQoL and depression with international validated questionnaires. The SF-12, an abridged version of the SF-36, is a reliable and valid tool for examining health-related quality of life, and has shown to be sensitive in various populations (22, 30). The EPDS remains an internationally widely applied reliable measure for depression (26). Maternal psychological outcomes are relevant for the treatment in the early postpartum period. The current study improves understanding of maternal HRQoL and depression after preterm births, especially in comparison with mothers of hospitalized term neonates, and enables a realistic assessment of the family's situation as well as allowing for improvements in clinical care.

This study was designed as a pilot study; therefore, it consists of quite a small number of participants, which might be a limiting factor for data interpretation. However, it can be assumed that the study cohort represents a sample of the standard population, as shown in the demographic data (see Table 1). Furthermore, patients with poor or absent German language abilities were excluded and might even have exacerbated the study results due to aggravated life conditions. A further larger study might verify our results and could focus on specific interventions and communication strategies in the NICU. Additional physical and psychological exams or inquiries would have enriched our findings. These might be topics for subsequent research.

### Implications for Practice

Obstetricians, neonatologists, clinical psychologists, and nurses are the primary experts providing support for women experiencing pregnancy complications leading to the need for their neonates' treatment in the neonatal care unit. In particular, extreme prematurity is accompanied by reduced mental HRQoL in the early postpartum period. Acknowledgment of concerns and fears and awareness of the needs of women may reduce prolonged psychological distress and improve self-efficacy for women and their families. Our findings specify that motherhood of preterm and full-term newborns requiring treatment in the neonatal ward is associated with high rates of depressive symptoms, reduced HRQoL and psychological distress. Adequate information and support from all health professionals in the obstetric and neonatal ward might be effective to reduce acute stress and anxiety. The goal of comprehensive care is to increase coping possibilities and resilience to reduce depression, anxiety and improve quality of life until the time of hospital discharge.

## Data Availability Statement

The raw data supporting the conclusions of this article will be made available by the authors, without undue reservation.

## Ethics Statement

The studies involving human participants were reviewed and approved by Ethics Commitee of the Medical University of Graz. The patients/participants provided their written informed consent to participate in this study. Written informed consent was obtained from the individual(s) for the publication of any potentially identifiable images or data included in this article.

## Author Contributions

EM, MD, EG, and AA: contributed substantial to conception and design, acquisition of data and analysis, and interpretation of data. EM, MD, EG, WS, AA, and CS: contributed to drafting the article and revising it critically for important intellectual content. All authors gave final approval of the version to be published.

## Conflict of Interest

The authors declare that the research was conducted in the absence of any commercial or financial relationships that could be construed as a potential conflict of interest.

## Publisher's Note

All claims expressed in this article are solely those of the authors and do not necessarily represent those of their affiliated organizations, or those of the publisher, the editors and the reviewers. Any product that may be evaluated in this article, or claim that may be made by its manufacturer, is not guaranteed or endorsed by the publisher.
